# Human Cytomegalovirus UL34 Early and late Proteins Are Essential for Viral Replication

**DOI:** 10.3390/v6020476

**Published:** 2014-01-28

**Authors:** Rico Rana, Bonita J. Biegalke

**Affiliations:** 1 Department of Biomedical Sciences, Heritage College of Osteopathic Medicine, Ohio University, 228 Irvine Hall, Athens, OH 45701, USA; E-Mail: rico.rana@yahoo.com; 2 Department of Biomedical Sciences and Program in Molecular and Cellular Biology, Heritage College of Osteopathic Medicine, Ohio University, 228 Irvine Hall, Athens, OH 45701, USA

**Keywords:** human cytomegalovirus, UL34, sequence-specific DNA-binding protein, nuclear localization

## Abstract

UL34 is one of the ~50 genes of human cytomegalovirus (HCMV) required for replication in cell culture in human fibroblasts. UL34 encodes highly related early (UL34a) and late (UL34b) proteins that are virtually identical, with the early protein containing an additional 21 amino terminal amino acids. The UL34 proteins are sequence-specific DNA‑binding proteins that localize to the nucleus. The HCMV genome contains 14 to 15 UL34 binding sites; two of the UL34 binding sites contribute to transcriptional regulation of two other viral genes, US3 and US9. The roles of the remaining binding sites and the requirement for both UL34 proteins during viral infection remain unknown. We examined the contributions of the early and late UL34 proteins to viral replication by generating HCMV-containing bacterial artificial chromosomes with the initiation codon for the early or the late protein mutated. Neither virus was able to replicate, demonstrating that UL34 expression is required throughout the viral replication cycle. A marked decrease in viral gene expression for each of the mutants suggests that UL34 proteins may contribute generally to transcriptional regulation. Intracellular localization studies demonstrated that UL34 colocalizes with the major immediate early protein, IE2, and the viral DNA polymerase processivity factor, UL44, to viral DNA replication centers. In conclusion, sustained UL34 protein expression is required for viral replication. The sequence-specific DNA binding ability of UL34 proteins, their localization to viral DNA replication centers and their general effects on viral gene expressions suggests that UL34 proteins contribute to the establishment of a nuclear environment necessary for viral gene expression and DNA replication.

## 1. Introduction

Human cytomegalovirus (HCMV) is predominantly an opportunistic pathogen, causing clinically significant disease in people who have inadequate immune responses, including neonates, transplant recipients, and people with uncontrolled HIV infections [1]. Viral replication initiates with the expression of the immediate early genes, the best characterized of which are IE1 and IE2. IE1 and IE2 initiate the cascade of viral gene expression, regulating the expression of the remainder of the viral genome, resulting in the expression of early and then late genes, and ultimately the production of new virions. 

HCMV has a very large genome of ~235 kb and encodes an estimated 176 genes [2]. Despite the large genome size, only ~50 genes are required for HCMV replication in cell culture, suggesting that the remaining 70% of the genes contribute to replication and latency in the human host [3,4]. The essential genes of HCMV can be grouped according to function: attachment of the virus to the target cells, transcriptional regulation, viral DNA replication, virion formation, and virion egress. Although there are a relatively small number of essential HCMV genes, to date the functions of several of the essential genes, including UL34, have been minimally characterized. 

The UL34 gene is essential for viral replication as determined by Dunn *et al.* [3] and Yu *et al*. [4] in their global analyses of the HCMV genome. The UL34 gene is transcribed throughout the viral replication cycle, resulting in the expression of early and late transcripts [5]. The early transcript becomes abundant by 3 hours post-infection (hpi) while the late transcript predominates from 48 hpi throughout the remainder of the viral replication cycle. Two highly related proteins are encoded by the early and late transcripts, with the late protein (UL34b) identical to the early protein (UL34a) except for the absence of 21 amino terminal amino acids. Both UL34 proteins localize to the nucleus and are sequence-specific DNA-binding proteins that act as transcriptional repressors; the interaction of UL34 proteins with the UL34 binding sites in the US3 and US9 genes down-regulates their expression [6,7]. 

In addition to the UL34 binding sites within the US3 and US9 genes, there are 12 to 13 additional binding sites located in the viral genome. Six of the UL34 binding sites are located within protein coding regions, 3 or 4 binding sites (the number is strain dependent) are in a region flanking the lytic origin of replication, and the remaining 3 binding sites are located 5' of protein coding regions [6]. The positions of the UL34 binding sites relative to coding regions suggests that UL34 proteins may be multifunctional, contributing not only to transcriptional repression but also contributing to viral replication in as yet unidentified ways. The experiments described here were undertaken to identify the contributions of each of the UL34 proteins to viral replication and to examine the intracellular localization pattern of UL34 proteins during infection. 

## 2. Results and Discussion

### 2.1. Both UL34 Proteins Are Essential for Viral Replication

Yu *et al.* [4] and Dunn *et al.* [3] identified UL34 as essential for viral replication in their global analyses of the HCMV genome. We extended their results by constructing and studying recombinant viruses using the bacterial artificial chromosome (BAC) that contains the HCMV AD169 genome, pHB5 [8]. HCMV-BACs that either entirely lacked UL34 (ΔUL34), contained UL34 with a mutation in the ATG initiating translation of the early protein [ATG mutated to ATC (methionine to isoleucine), ΔE mutant], contained UL34 with a mutation in the ATG initiating the late protein [ATG mutated to GTG (methionine to valine), ΔL mutant], or had the UL34 open reading frame restored (UL34 rescue, RUL34). The ability of each of the recombinant viruses to replicate was assayed following electroporation of the HCMV-BACs into primary human fibroblasts, along with a plasmid expressing the tegument protein, pp71. Following electroporation, cells were observed for plaque formation for 4 weeks. The parental BAC, pHB5, and the UL34 rescue BAC (RUL34) gave rise to plaques by 8 days post-transfection. No plaques developed in the cells receiving the ΔUL34 mutant, the ΔE UL34 mutant or the ΔL UL34 mutant BAC during a 4 week observation period. From these results, we concluded that the expression of both UL34 proteins is essential for viral replication. 

### 2.2. Reduced Viral Gene Expression in the Absence of UL34 Proteins

**Figure 1:**
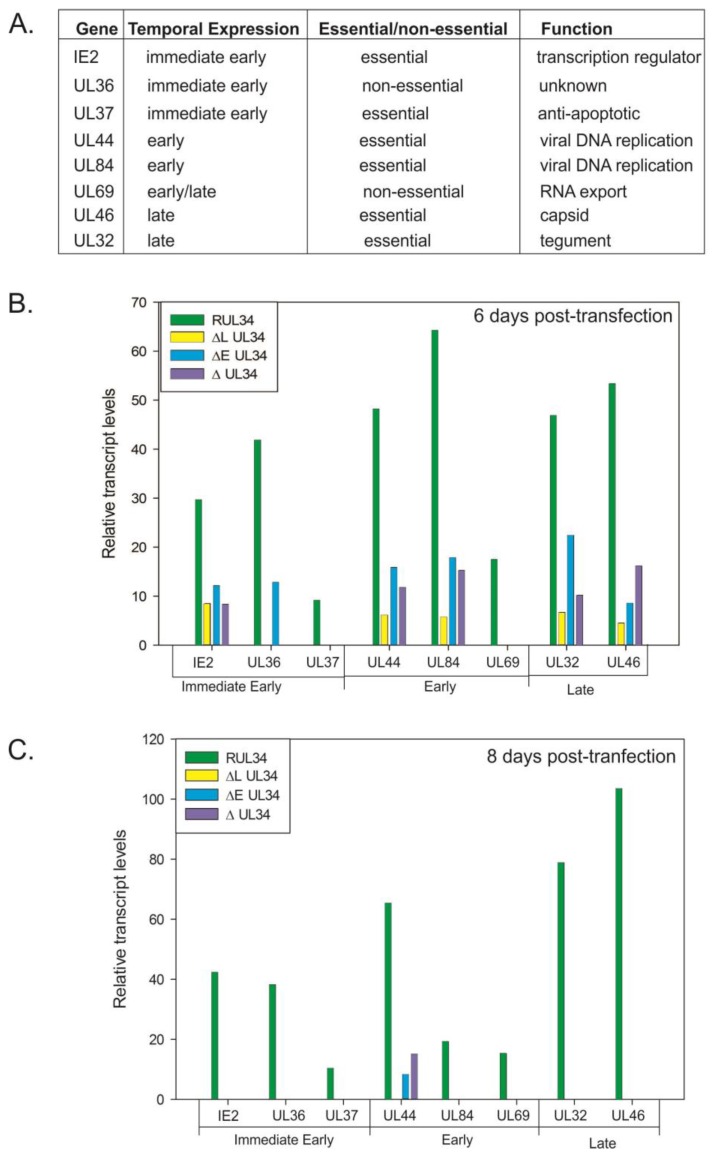
(**A**) List of genes assayed for expression in cells receiving the recombinant UL34 human cytomegalovirus (HCMV) bacterial artificial chromosome (BACs). (**B**) and (**C**) Relative transcript levels for the indicated genes at 6 and 8 days post-transfection. RT-PCR was used to amplify the transcripts for each of the listed genes along with the cellular gene, glyceraldehyde phosphate dehydrogenase (GAPDH). The amplification products were quantified; viral gene levels were normalized to the level of GAPDH amplimers obtained for each of the samples. RUL34 is the UL34 rescued HCMV BAC, ΔL UL34 has the initiation codon for the late protein mutated, ΔE UL34 has the initiation codon for the early protein mutated and ΔUL34 has the entire UL34 open reading frame deleted.

To examine the defect in viral replication associated with the absence of UL34 proteins, semi-quantitative RT-PCR reactions were performed on RNA samples extracted following the electroporation of the UL34-HCMV BACs into human fibroblasts. Levels of expression for the essential genes UL32, UL37, UL44, UL46, UL84 and UL123 (IE2) were assayed as were levels of expression of the non-essential UL36 and UL69 genes. IE2, UL36, and UL37 are immediate early genes; UL44 and UL84 are early genes; UL69 is an early/late gene and UL32 and UL46 are late or presumed late genes (Figure 1A). Levels of expression were analyzed at 6 and 8 days post-transfection; time points that correspond approximately to early and late times of infection, based on the time when plaques are visible. Viral transcript levels were normalized to the transcript levels of the cellular gene, glyceraldehyde phosphate dehydrogenase (GAPDH). At 6 and 8 days post-transfection, transcript levels for all genes asssayed were decreased in the UL34 mutant viruses when compared to the UL34 rescued virus (RUL34, Figure 1B,C). 

Six days post-transfection, expression of the major immediate early (mIE) gene, IE2, was decreased in the absence of the early, late or both UL34 proteins. Similar to the reduction in the level of IE2 transcripts, levels of UL44, UL84, UL32, and UL46 transcripts were detected at a reduced level for all of the UL34 mutant viruses at 6 days post-transfection (Figure 1B). In contrast, no UL69 or UL37 expression was detected for the UL34 mutant viruses; and UL36 expression was detected only in the UL34 mutant virus expressing the late UL34 protein (UL34b). At 8 days post-transfection, only UL44 transcripts were detectable for the UL34 deleted-BACs, albeit at a much reduced level compared to the UL34 rescued virus (Figure 1C). 

There is no UL34 binding site within the major immediate early gene, and in studies utilizing transient expression assays, UL34 has no activating or repressing effect on the mIE promoter [9]. Consequently, the reduction in IE2 transcript levels seen in the absence of UL34 proteins suggests that UL34 proteins have a general effect on the level of IE2 transcripts. This is supported by the reduction in transcript levels for all viral genes assayed (Figure 1B,C). UL32 and UL37 contain UL34 binding sites within their open reading frames; however, the diminution in transcript levels is consistent for all genes tested, regardless of the presence of a UL34 binding site. Intriguingly, the inability to detect UL69 and UL37 transcripts in the absence of either UL34 protein suggests that both proteins are required for their expression, contrasting with UL36 expression, where transcripts are not detectable in the absence of the late UL34 protein, suggesting that the late UL34 protein (UL34b) is required for UL36 expression.

At 8 days post-transfection, of the genes assayed, only UL44 transcripts were detected for the UL34 mutant viruses, in contrast to the transcripts detected for the UL34 rescued virus (Figure 1C). The lack of either UL34 protein results in a decrease in expression of IE2 and an absence of UL37 expression; proteins critical for later gene expression. These data suggested that the effects of the UL34 mutations were cumulative, that is, a reduction in the expression of essential genes earlier in the infection cycle (day 6) results in very little viral gene expression by 8 days post-transfection, corresponding to the defect in viral replication. 

Some variations in transcript levels were seen when comparing the UL34 mutant HCMV BACs. Expression of only the late UL34 protein resulted in detectable levels of UL36 and an increase in the level of UL32 expression, relative to the other UL34 mutant viruses, suggesting that the late protein plays a significant role in the expression of these two genes. There are UL34 binding sites located within the coding regions of UL37 and UL32 that may directly influence gene expression. UL34 proteins have a known transcriptional effect, repressing expression of the US3 and US9 genes [6,7]. However, the data presented in Figure 1 suggests that UL34 has a more general effect on viral gene expression given the lack of UL34 binding sites within the other assayed genes, coupled with the decrease in gene expression. Both forms of the UL34 protein are required for viral replication, with the early and late proteins differentially affecting the expression of other viral genes.

### 2.3. UL34 Localizes to Viral DNA Replication Centers

The UL34 proteins localize to the nucleus when visualized using a tag of enhanced green fluorescent protein (EGFP) or using indirect immunofluorescence [5]. Here, we examined the intracellular location of UL34 proteins during the viral replication cycle, using polyclonal rabbit anti‑sera to UL34 and indirect immunofluorescence. Monoclonal antibodies to other viral proteins were used simultaneously to co-label the same infected cell population. Primary human fibroblasts were infected with HCMV strain Towne and fixed and stained at 3, 24, 48, 72 and 96 hours post‑infection (hpi). 

**Figure 2:**
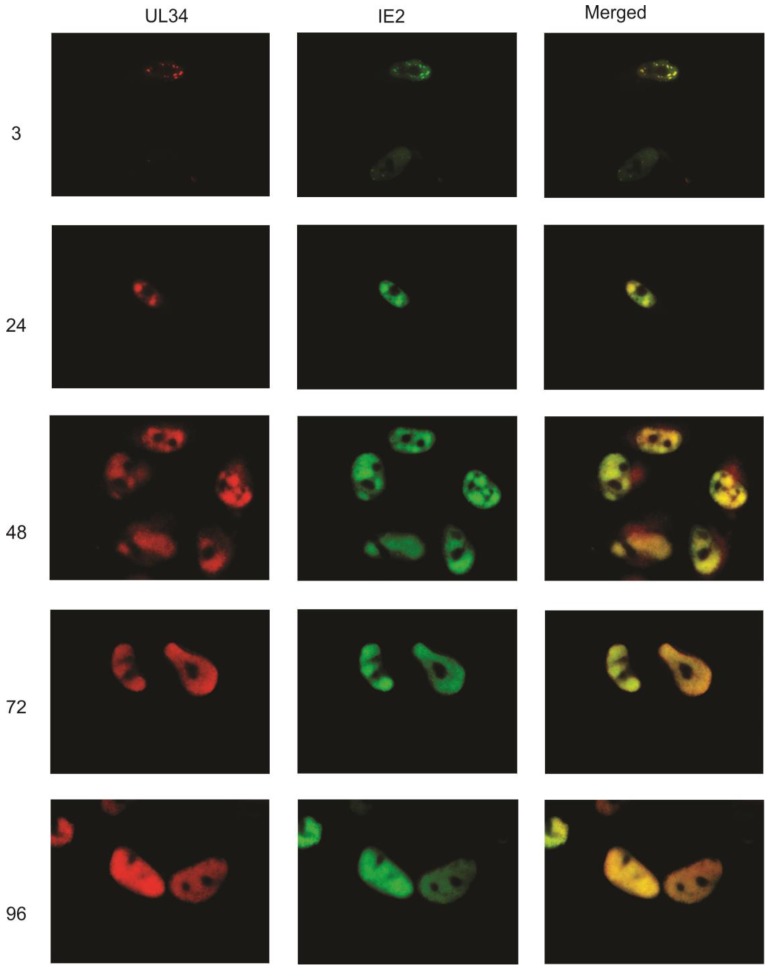
Indirect immunofluorescence was performed on primary human fibroblasts infected with HCMV strain Towne at 3, 24, 48, 72, and 96 hpi using antibodies to UL34 and to IE2. UL34 staining is red, IE2 staining is green; the merged column is the combination of the green and red images. Colocalization is indicated by the yellow color in the merged images. The numbers indicate the time post-infection at which the cells were examined. Control slides, infected cells incubated only with secondary antibodies, are not shown.

As UL34 proteins are detectable by 4 hpi using either immunoprecipitation or western blot analyses [5], we initially compared the intracellular pattern of UL34 expression to that of the immediate early protein, IE2. At 3 hpi, UL34 was detected in a punctate staining pattern (Figure 2). Co-labeling with an antibody to IE2 demonstrated that UL34 colocalized with IE2 in the punctate dots at 3 hpi. UL34 continued to colocalize with IE2 throughout the remainder of the viral replication cycle, with the staining of UL34 and IE2 increasing as viral replication progressed (Figure 2). At 3 hpi, UL34 was not detectable in all cells positive for IE2, as expected, with IE2 expression activating the expression of other viral genes. 

**Figure 3:**
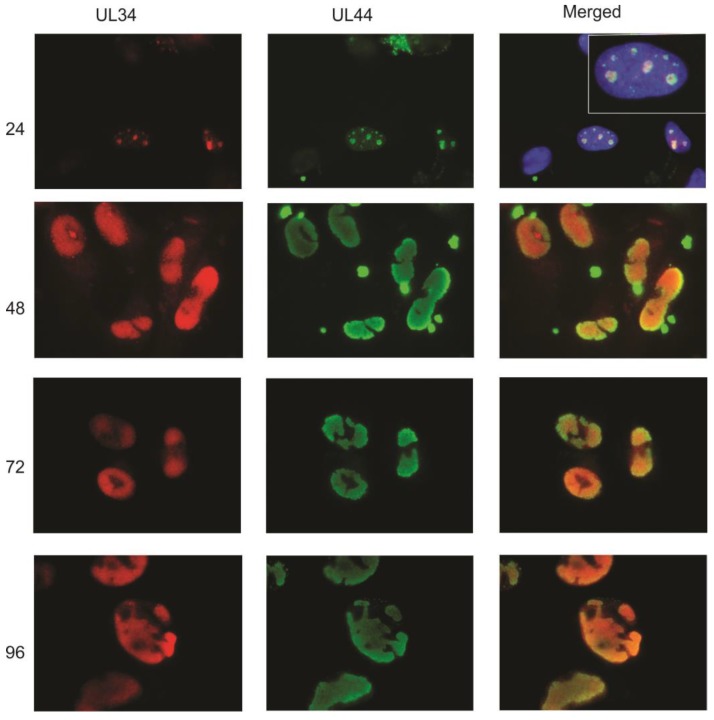
Indirect immunofluorescence was performed on primary human fibroblasts infected with HCMV strain Towne at 24, 48, 72, and 96 hpi using antibodies to UL34 and to UL44. UL34 staining is red, UL44 staining is green; the green and red images were merged with the DAPI-stained images (blue) at 24 hpi; for the other timepoints, only the red and green images were merged. An enlargement of one of the stained cells is shown in the insert at 24 hpi in the merged column. The extracellular bright green dots seen in the 48 hpi samples were an artifact associated with the secondary antibody.

As IE2 is found in viral DNA replication structures [10], the colocalization of UL34 with IE2 suggested that UL34 localizes to viral DNA replication centers. To further examine the intracellular location of UL34, additional immunofluorescence experiments were performed, comparing the intracellular location of UL34 to that of UL44. UL44 encodes a viral DNA polymerase processivity factor; antibody labeling of UL44 is commonly used to identify viral DNA replication centers [11,12]. UL44 was detectable at 24 hours post-infection, localizing as bright nuclear foci with a low level of diffuse nuclear staining (Figure 3). UL34 colocalized with UL44 in the bright nuclear foci. The pattern of colocalization continued throughout the viral replication cycle, with UL34 colocalizing extensively with UL44. Although UL34 colocalizes with UL44 in viral DNA replication centers, UL34 was not identified in the proteins complexing with UL44 [11], suggesting that UL34 does not interact directly with UL44.

**Figure 4:**
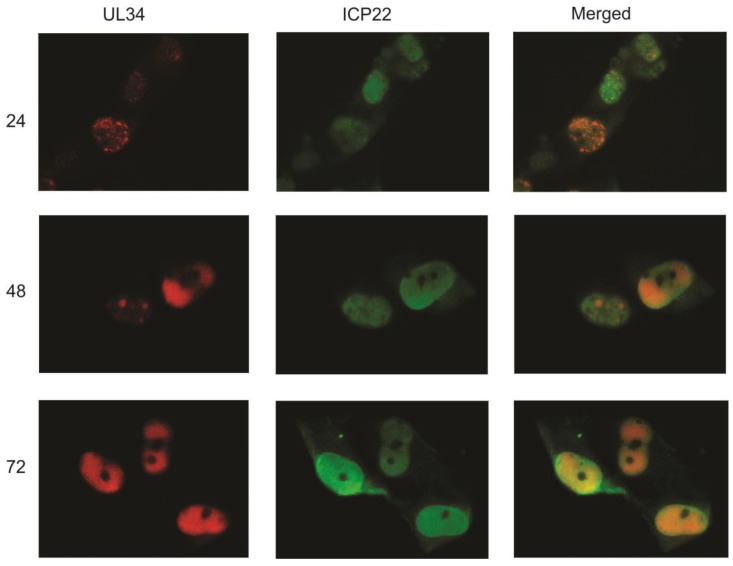
Indirect immunofluorescence was performed on primary human fibroblasts infected with HCMV strain Towne at 24, 48, and 72 hpi using antibodies to UL34 and to ICP22. UL34 staining is red, ICP22 staining is green; the merged column is a combination of the red and green images.

The similar staining patterns of UL34, IE2 and UL44 again suggested that UL34 localizes predominantly to viral replication centers. To compare the intracellular location of UL34 with that of another viral protein found in the nucleus but not in viral DNA replication centers, infected cells were labeled with antisera to UL34 and with a monoclonal antibody to ICP22. The US22 gene encodes ICP22, a tegument protein that is found in the nucleus early in infection and in both the nucleus and cytoplasm at later stages of viral replication [13]. As shown in Figure 4, little colocalization of UL34 with ICP22 is seen, suggesting that UL34 specifically colocalizes with IE2 and UL44 in viral DNA replication centers.

IE2 accumulates at the periphery of promyelocytic leukemia protein-associated nuclear bodies (PODs) [10], becoming incorporated into viral DNA replication centers, along with the core proteins including UL44, as the viral replication cycle continues. Our data demonstrate that UL34, along with IE2 and UL44, is also found in viral DNA replication centers. The contribution of UL34 to viral replication is potentially mediated through the UL34 binding sites located in the viral genome near the origin of lytic replication. The interaction of pUL34 with the ori-lyt region and the localization of UL34 to viral DNA replication center along with the two essential proteins, IE2 and UL44, suggests that UL34 may contribute to the efficiency of viral DNA replication. 

### 2.4. UL34 Colocalizes with Nucleolin

**Figure 5:**
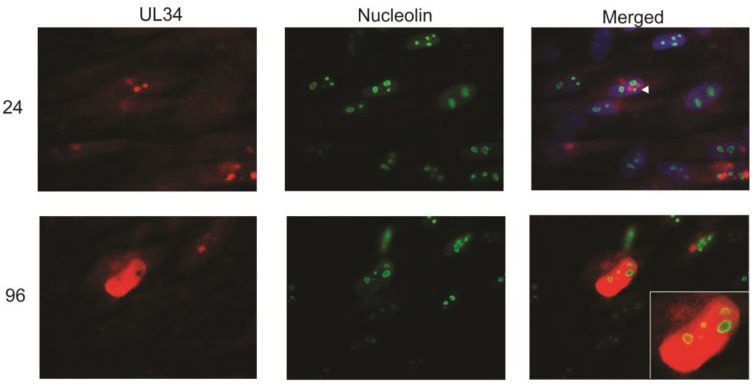
Indirect immunofluorescence was performed on primary human fibroblasts infected with HCMV strain Towne at 24 and 96 hpi using antibodies to UL34 and to nucleolin. UL34 staining is red, nucleolin staining is green; the green and red images were merged with the DAPI-stained images (blue) in the merged column at 24 hpi as described for Figure 1; the DAPI-stained image was not included in the merged image for the 96 hpi timepoint. The arrowhead indicates the association of UL34 with nucleolin early in infection. The inset shows an enlargement of the colocalization of UL34 and nucleolin at 96 hpi.

At late stages of infection (96 hpi), UL34 was found widely distributed in the nucleus. The circular areas within the nucleus that did not contain UL34 (See Figure 2, 72 hpi as an example) were identified as nucleoli using an antibody to label the nucleolar protein, fibrillarin (data not shown). However, UL44 colocalizes partially with the cellular protein nucleolin, and the interaction of nucleolin with UL44 is essential for viral replication [11]. The colocalization of UL34 and UL44 suggested that UL34 would colocalize with nucleolin. To examine the interaction of UL34 and nucleolin, infected cells were costained with antibodies to UL34 and to nucleolin. Early in infection (24 hpi), UL34 accumulated in bright nuclear foci adjacent to the nucleolin staining (Figure 5). However, at late times of infection, UL34 partially colocalized with the ring of nucleolin detected at the perimeter of the nucleoli (Figure 5, see inset). These data suggested that nucleolin and nucleoli contribute to organization of UL34 in the nucleus and to the formation and organization of viral DNA replication compartments. Strang *et al*. [11,12] found nucleolin predominantly dispersed in the nucleus following viral infection (using HCMV strain AD169), and further found that UL44 partially colocalized with nucleolin at the perimeter of viral replication centers. In contrast, using a monoclonal antibody to nucleolin rather than polyclonal antisera, we detected no dispersal of nucleolin. The difference seen in nucleolin distribution following infection may be a result of utilizing different antisera, or alternatively, strain specific differences, with the localization data presented here obtained following infection with HCMV strain Towne. In summary, UL34 proteins are localized in close proximity to nucleolin early in infection; late in infection, UL34 proteins partially colocalize with nucleolin.

## 3. Experimental Section

### 3.1. Virus, Cells and Immunofluorescence

HCMV strain Towne was propagated in primary human fibroblasts in Dulbecco’s minimal essential medium supplemented with glutamine, penicillin, streptomycin and 10% Nuserum. For immunofluorescence studies, primary human fibroblasts were plated on glass coverslips and infected with HCMV strain Towne at a moi of 1–2 pfu/cell. Prior to antibody staining, cells were washed with phosphate buffered saline (PBS), fixed with 4% paraformaldehyde and permeabilized using 0.2% Triton X-100 in PBS. Following four washes with PBS, cells were incubated with 10% normal human serum at 37 °C for 1 hour. Primary antibodies were diluted to the recommended concentrations in PBS containing 3% bovine serum albumin; cells were incubated with the primary antibodies for 1 hour at room temperature. Secondary fluorescent dye-tagged antibodies to mouse or rabbit antibodies were incubated with the cells for 30 minutes at room temperature. Nuclei were stained with 4',6-diamidino-2-phenylindole (DAPI); cells were visualized using a Nikon fluorescence microscope and photographed using a SPOT camera. The following primary antibodies were used: monoclonal antibodies to UL44 (ICP36) and to US22 (ICP22) were obtained from Virusys Corporation, Taneytown, MD, USA; the monoclonal antibody to IE2 was obtained from Millipore, Billerica, MA, USA; the monoclonal antibody to nucleolin was obtained from Abcam, Cambridge, MA, USA, and the fluorescent dye-tagged secondary antibodies were obtained from Life Technologies, Grand Island, NY, USA.

### 3.2. Recombinant Virus

The BAC plasmid, pHB5, which contains the HCMV AD169 genome was kindly provided by U. Koszinowski [8]. A mutated BAC lacking UL34 was constructed as described by Datsenko and Wanner [14] using primers (285, 5'CAGAACCCGTCGCCATTTCCCCTCATA-TACGGTACACGTCCCC CTGATCTGTGTAGGCTGGAGCTGCTTC 3' and 286, 5' AAACCAGAGCGGAACTTGAGAAATCAACGCTTTATTGTTCTCCAGTGACGCATAT-GAATATCCTCCTTAG 3') which contain sequences complementary to the ends of UL34 and to the kanamycin gene [14]. Briefly, the primers were used to amplify the kanamycin and frt sequences from pKD13; the PCR product was then introduced into pHB5 using pKD46, which expresses the lambda Red genes. Following insertion of the FRT-kanamycin cassette and deletion of the UL34 open reading frame, the kanamycin gene was removed from the BAC using pCP20 which expresses the Flp recombinase. 

Recombinant BACs were then constructed by replacing the deleted UL34 gene with either the wild type sequence or with a UL34 gene containing mutations of either the early or late translation initiation codons for the early and the late proteins. pBJ545, which contains the HindIII H fragment of HCMV strain Towne in pST76K_SR, was mutated using oligo pairs 328 and 329 (328, 5' CCTGCGAGCCGCCGAGGTGCGTGACAACGTGGC 3'; 329, 5' GCCACGTTGTCACGC ACCTCGGCGGCTCGCAGG 3') or 330 and 331 (330, 5' ACCGCCCCACCGCCGTCGTCGTCAT CAACTTCATCATCACCACC 3'; 331, 5' GGTGGTGATGATGAAGTTGATGACGACGAC GGCGGTGGGGCGGT 3') and the Quickchange mutagenesis kit (Stratagene, Santa Clara, CA, USA) giving rise to pBJ598 (early ATG mutated to ATC) and pBJ599 (late ATG mutated to GTG). pBJ598 and 599 were electroporated into bacteria containing the ΔUL34-BAC and pKD46. Resulting colonies were screened using PCR to identify BACs containing the replaced UL34 sequences. 

### 3.3. Quantitative Reverse Transcriptase Polymerase Chain Reactions

The following pairs of oligonucleotides were used to amplify a portion of the indicated gene transcript: UL37 (368, 5' TCAGACGATCCGATGAACGT 3' and 369, 5' TCTCCTCCGAGCCAAAAGTC 3'), UL44 (370, 5' CTAGCCGCACTTTTGCTTCT 3'and 371, 5' ACGGTCTTTCCTCCAAGGAA 3'), UL69 (372, TTAGTCATCCATATCATCGC 3' and 373, GAGCTTAACTTGATGACGCC 3'), UL122 (374, GAGACTTGTTCCTCAGGTCC 3', and 375, 5' CAACATGATCATCCACGCTG 3'), UL36 (376, 5' TCAGTTGTTCATGTAAACGT 3', and 377, ACCACTTTGAACTCTCCTAC 3'), glyceraldehyde phosphate dehydrogenase (GAPDH; 39, 5‘ AGAGACATCATCCCTGCCTCT 3', and 391, 5' TTTTTCTAGACGGCAGGTCA 3'), UL84 (398; 5' GCAGACCATGGCTAAAGTGA 3' and 399, 5' TTAACCGTACTGGTGAGCGA 3') and UL32 (20, 5' TGCAGTTTATCGGTCTACAGCG 3'; and 21, 5'CGRATCCTTGAGGTGCACAAAG 3'). RNA was extracted from human fibroblasts that had been electroporated with the indicated HCMV-BAC 6 and 8 days post-transfection. RNA was treated with RQ1 DNAse followed by DNAse stop solution. PCR reactions were performed following reverse transcription reactions or in the absence of reverse transcription to confirm the absence of contaminating DNA. Amplimers were quantified following gel electrophoresis.

## 4. Conclusions

The experimental results presented here demonstrate that both UL34 proteins play an essential role in viral replication. Viruses containing in frame point mutations substituted for the initiation codons for the early and the late proteins are replication defective. The absence of UL34 protein expression resulted in a diminution of viral gene expression, and most notably, the absence of UL37 and UL69 transcripts. With the exception of UL34 repression of US3 and US9 expression [6,7], we have been unable to identify any direct effects of UL34 expression on viral gene transcription. This suggests that UL34 proteins are multi-functional, not only specifically repressing the expression of some viral genes, but also having a general effect on viral gene transcription. We postulate that the effects of the UL34 proteins may be to facilitate the establishment of an environment in the nucleus favorable for viral gene expression and viral DNA replication. This notion is supported by the localization of UL34 proteins to viral DNA replication centers. Furthermore, the proximal localization of UL34 to nucleolin suggests that UL34-nucleolin interactions may facilitate the intranuclear positioning of the replication centers. In summary, this work demonstrates the significance of both UL34 proteins, and raises questions about the viral and cellular proteins that UL34 proteins interact with.
